# Conductive Paper with Antibody-Like Film for Electrical Readings of Biomolecules

**DOI:** 10.1038/srep26132

**Published:** 2016-05-23

**Authors:** Ana P. M. Tavares, Nádia S. Ferreira, Liliana A. A. N. A. Truta, M. Goreti F. Sales

**Affiliations:** 1 BioMark-CINTESIS/ISEP, School of Engineering, Polytechnic Institute of Porto, Portugal

## Abstract

This work reports a novel way of producing an inexpensive substrate support to assemble a sensing film, designed for the electrical transduction of an intended biomolecule. The support uses cellulose paper as substrate, made hydrophobic with solid wax and covered by a home-made conductive ink having graphite as core material. The hydrophobicity of the paper was confirmed by contact angle measurements and the conductive ink composition was optimized with regard to its adhesion, conductivity, and thermal stability. This support was further modified targeting its application in quantitative analysis. Carnitine (CRT) was selected as target compound, a cancer biomarker. The recognition material consisted of an antibody-like receptor film for CRT, tailored on the support and prepared by electrically-sustained polymerization of 3,4-ethylenedioxythiophene (EDOT) or dodecylbenzenesulfonic acid (DBS). Fourier transform infrared spectroscopy (FTIR) and Raman spectroscopy analysis confirmed the presence of the polymeric film on the support, and the performance of the devices was extensively evaluated with regard to linear response ranges, selectivity, applicability, and reusability. Overall, the paper-based sensors offer simplicity of fabrication, low cost and excellent reusability features. The design could also be extended to other applications in electrical-based approaches to be used in point-of-care (POC).

A medical decision is often supported by laboratorial tests. With the exception of pregnancy and glucose measurements, these tests are performed at hospital or independent laboratories (including large reference laboratories). This procedure takes time and may be at the borderline between life and death, while requiring significant economic/human resources. Point-of-care (POC) analysis is therefore considered an important tool in clinical context. POC testing can also be more accurate by avoiding analyte changes during sample transport/storage, caused by delayed release of analytes (e.g., release of K^+^ from red blood cells during refrigerated storage), by continued metabolism (e.g., decrease in glucose/pH and increase in lactate from active red blood cells under hypoxic conditions), and by protein/peptide degradation in whole blood^1^,^2^.

The devices involved in POC testing should be portable, small, easy to use and carry, and inexpensive^3^,^4^. The combination of immunoreactions with compact apparatus (for signal reading) and microfluidic units (for sample handling) is today the closer approach meeting POC requirements. Immunological-based methods such as enzyme-linked immune sorbent assays (ELISAs) can detect low concentrations of several chemical and biological analytes in biological samples^5^,^6^. The experimental tests may take however longer than desired and require qualified personnel. In addition, these methods may not be sensitive enough to screen a wide range of proteins in low concentrations^7^,^8^,^9^.

In alternative of these systems, biosensors have emerged several years ago to reproduce immunoreactions (or other biological reactions) on a flat receptor support, becoming more sensitive and simpler. This approach also follows the advances made in glucose readings, this time making use of enzymatic reactions. In general, a biosensor is a self-contained integrated device that uses biological molecules as recognition element, in direct contact with a transducer of optical, mass, magnetic or electrical nature^10^.

Accounting the emerging advances in nanotechnology, biosensors also offer the possibility of easy miniaturization. In this regard, electrical biosensors (also known as electrochemical devices) have shown important developments, mostly related to the concomitant advances in screen-printing technology^11^. The resulting screen-printed electrodes (SPEs) combine in a single support all electrodes required for electrochemical readings: work, auxiliary and reference electrodes. Although many advantages have been achieved in this course, several requirements need to be met at their mass production^12^, and their commercialization is still expensive. In addition, their specific use in POC diagnosis should be linked to a disposable device of little environmental impact. Yet, printed supports are mostly plastics, such as PET or PVC. Thus, a worldwide application of such devices requires replacing such synthetic materials. Paper is one of several possibilities in this context, offering the advantages of being readily available.

In addition to the support, the recognition element is a fundamental component to reach the desired sensitivity and selectivity. So far, commercially available devices employ recognition elements of biological origin. But receptors like enzymes and antibodies have limited stability and cannot be used under harsh conditions. The search for more stable synthetic materials mimicking highly selective and sensitive recognition processes occurring in nature is therefore of interest to the biomedical field. Nature’s selectivity and sensitivity have not yet been matched, but steady progresses are being made in creating synthetic systems for molecular recognition.

The design of plastic antibodies by molecular imprinting (MI) technology is one possible approach to replace biological receptors. These biomimetic materials may be assembled in the laboratory, with several monomeric pieces, targeting almost any compound of interest. This can be done by bulk imprinting, where the target compound is mixed with the monomers, or by surface imprinting^13^, where the target compound is attached to a support by covalent bonding^14^ or adsorption^15^. Electropolymerization may also be employed, where the polymerization is initiated by an electrical stimulus^15^. All these are suitable routes for a successful production of synthetic receptors, mimicking natural antibodies.

These combined approaches have been tried out herein for carnitine (CRT). CRT, 3-hydroxy-4-*N,N,N*-trimethylaminobutyric acid, is a biogenic quaternary amine, playing an essential role in the mitochondrial β-oxidation of long-chain fatty acids^16^. Besides its important role in cellular metabolism and energy production, CRT also displays antioxidant properties, protecting cells from oxidative stress conditions related to several diseases^17^,^18^,^19^. In addition, CRT is a potential biomarker of ovarian cancer, because it was identified as a metabolite in the normal ovary and transformed in primary and metastatic ovarian cancer^20^,^21^. This is particularly relevant because ovarian cancer is the seventh most common cancer in women worldwide and causes more deaths than any other gynecologic malignancy, being diagnosed mostly at an advanced stage^21^,^22^. There are several analytical methods to determine CRT in several samples, suitably reviewed^23^,^24^,^25^. Few of these methods offer portability features and the ones nearing it do not combine disposable supports with stable biorecognition materials, which are essential conditions to promote worldwide POC applications in early screening of ovarian cancer.

Thus, the construction of a novel, disposable, stable and low cost device, with an antibody-like film assembled on paper-based conductive supports is presented herein. For this purpose, a regular paper is made hydrophobic and covered by a conductive ink. The doctor Blade technique was used for this purpose. The conductive ink proved good thermal/electrical stability, good conductivity and reusing feasibility, not easily removed after subsequent washing. The antibody-like film was assembled by polymerizing EDOT or DBS on the conductive support and in the presence of CRT. The biomimetic surfaces so-obtained were evaluated by electrochemical techniques and surface analysis, displaying successful features to be applied to the analysis of biological samples.

## Results and Discussion

**Figure 1:**
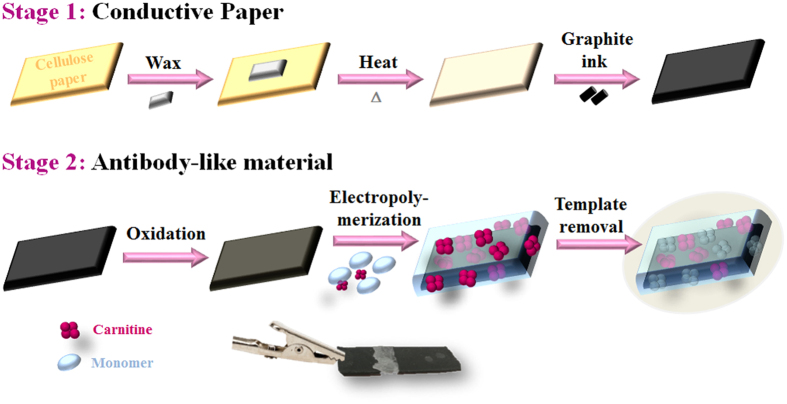
Schematic representation of the production of the conductive paper and its sensitization by an antibody-like material.

The production of a conductive paper involved two different stages (Fig. 1). The first one considered a pre-treatment of the paper to turn cellulose supports hydrophobic and the second one producing a suitable homemade conductive ink to cover such hydrophobic support. Only after this stage the antibody-like film was assembled on the material, as described later.

### Paper pre-treatment

**Figure 2:**
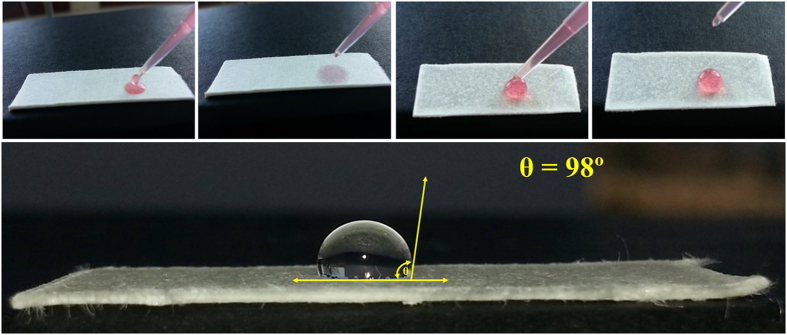
Picture of hydrophilic (**A**) and hydrophobic paper (**B**) with a drop of a colored aqueous solution on top for 0 (A_1_/B_1_) and 1 minute (A_2_/B_2_), and the subsequent contact angle of the hydrophobic paper (**C**).

Cellulose is a biodegradable and renewable resource of well-known hydrophilic features^26^, absorbing rapidly any drop of an aqueous solution placed on its surface (as shown in Fig. 2A). But the use of paper as support for electrical readings does not allow the existence of interactions between water molecules and the β-(1→4)-glucose polymeric structure, because these interactions interfere with the electrical properties of the resulting surface. To avoid such interactions, the paper was made hydrophobic by covering the cellulose fibres with a fat compound. Wax was selected for this purpose, because it becomes liquid after moderate heating and may interpenetrate all cellulose fibres with high efficiency. Once cooled to room temperature, the oily compound became solid and remained on its position, covering all cellulose fibres and yielding a paper/wax matrix.

In this work, each piece of paper was placed individually inside an oven, with specific amounts of wax on top of it, and heated for specific periods of time and temperature ranges. The best condition was found by monitoring the water-resistant properties of the final conductive paper inside the measuring buffer solution. Overall, an amount of 55 g wax per 56 mm^3^ piece of paper gave rise to a paper/wax matrix that repelled water, as observed on Fig. 2B. The aqueous solution was no longer absorbed on the paper and remained at the surface.

The hydrophobic pattern and the wettability of the wax paper were evaluated by contact angle measurements^27^. Contact angles reflected how strongly the liquid and the solid molecules interact with each other; when the molecules of an aqueous solution are weakly attached to the molecules at a solid surface, a drop of that solution will remain on its surface and the contact angle formed by it will depend on the extension of such attractive/repealing forces^28^. In general, angle values above 90° confirm the hydrophobic character of a given surface, with poor wetting properties and poor adhesiveness to liquid substances. In the present study, the water drop on the paper/wax presented a contact angle of 98° (Fig. 2C), thereby confirming its hydrophobic character and its possible application in electrical transduction systems.

**Figure 3:**
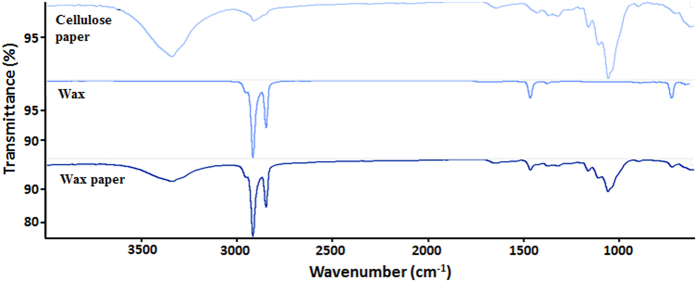
FTIR spectra of several materials at different stages of the production of conductive paper.

The presence of wax within the cellulose fibers was also confirmed by FTIR studies (Fig. 3). As expected, the cellulose paper showed a broad band centered at 3342.41 cm^−1^, corresponding to the O–H stretch, and a peak at 1161.18 cm^−1^, characteristic of the C–O stretch, both bonds intensely present at the glucose sub-units of cellulose. The wax spectra evidenced the C–H stretch typical of alkane compounds at 2849.18 and 2917.09 cm^−1^ and a small intensity peak at 721.79 cm^−1^, corresponding to consecutive –CH_2_– groups characteristically present in paraffin. The FTIR spectra combined all the previous peaks, thereby confirming the successful inclusion of wax among the cellulose fibers.

### Turning the paper conductive

A carbon-based ink made of graphite was prepared to confer electron conduction properties to the wax/paper support. Graphite has good electrical features, being readily available at a low cost.

It can also improve the accumulation of a target molecule at the electrode surface by electrochemical adsorption from any solution^29^. PVC-COOH was selected as polymeric support of the graphitic structures dispersed through the final ink. Thus, co-dissolution of graphite and PVC-COOH was tried out with different solvents. The best compromise between the viscosity of the ink solution and stability/conductivity of the final material was obtained by using DMF solutions. Since PVC-COOH is a non-conductive material, it is expected to increase the Ohmic resistance of the final ink and therefore its quantity should be set to a minimum value. On the contrary, the adhesion of the ink to the wax-paper support would be improved by increasing the concentration of PVC-COOH. Thus, ink solutions were prepared with varying amounts of PVC-COOH: 5, 15, 20, 25 and 30%. The corresponding solutions were applied by following the doctor Blade technique and the papers let dry in an oven for 1 hour.

The resulting ink-paper resistance ranged 1.0–1.7; 0.9–1.5; 0.8–2.0; 7–11; and 2.0–28 KΩ, respectively. These values were obtained by measuring the final resistance at several spots within each paper and in different papers produced in the same way. In general, and as expected, the conductivity values increased with the decreasing amount of PVC-COOH. Ink solutions with 5% polymer gave rise to conductive wax papers of low conductivity values, but the final surface was heterogeneous and the graphitic structures pealed out easily from the surface by physical contact. Above 20% PVC-COOH, the dry ink was very stable against external damage, but displayed poor conductivity.

**Figure 4:**
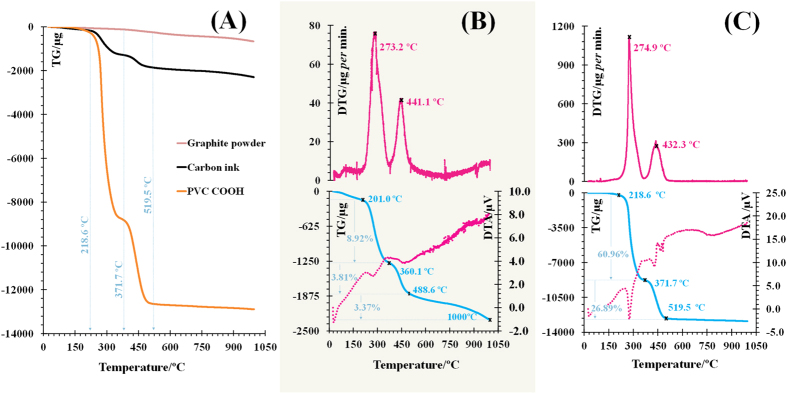
Thermogravimetric plot of conductive ink, pure graphite and pure PVC-COOH (**A**), with the corresponding differential data, DTG/DTA (Carbon in (**B**) and PVC-COOH in (**C**).

So, the final ink was prepared with 85% graphite and 15% PVC-COOH, co-dissolved in DMF. The thermal stability features of this ink (after drying) were evaluated by measuring mass loss curves (TG) and their first derivatives (DTG and DTA) compared to control materials, up to 1000 °C. The resulting thermogram is shown in Fig. 4A. It displays the behaviour of single graphite and PVC-COOH under thermal decomposition and their combined action when mixed to produce a conductive ink. The most significant decomposition observed accounted mostly the presence of PVC-COOH, starting at 218.6 °C, and ending at 519.5 °C. Ink mass losses up to 488.6 °C (Fig. 4B) were in good agreement with the % of PCV-COOH present in the ink. The total % ink mass loss was 12.73%, which corresponded to 85% of its initial mass (15%), while pure PVC-COOH had mass losses of 88%, up to 520 °C (Fig. 4C). These results suggested that no chemical interaction was established between the two ink ingredients. An additional ink mass loss of 3.37% was observed before 1000 °C, which corresponded to mass losses occurring similarly in pure graphite. The results also showed that the ink was thermally stable up to 200 °C. Additional discussion regarding thermal analysis data is presented in the supplementary section.

**Figure 5:**
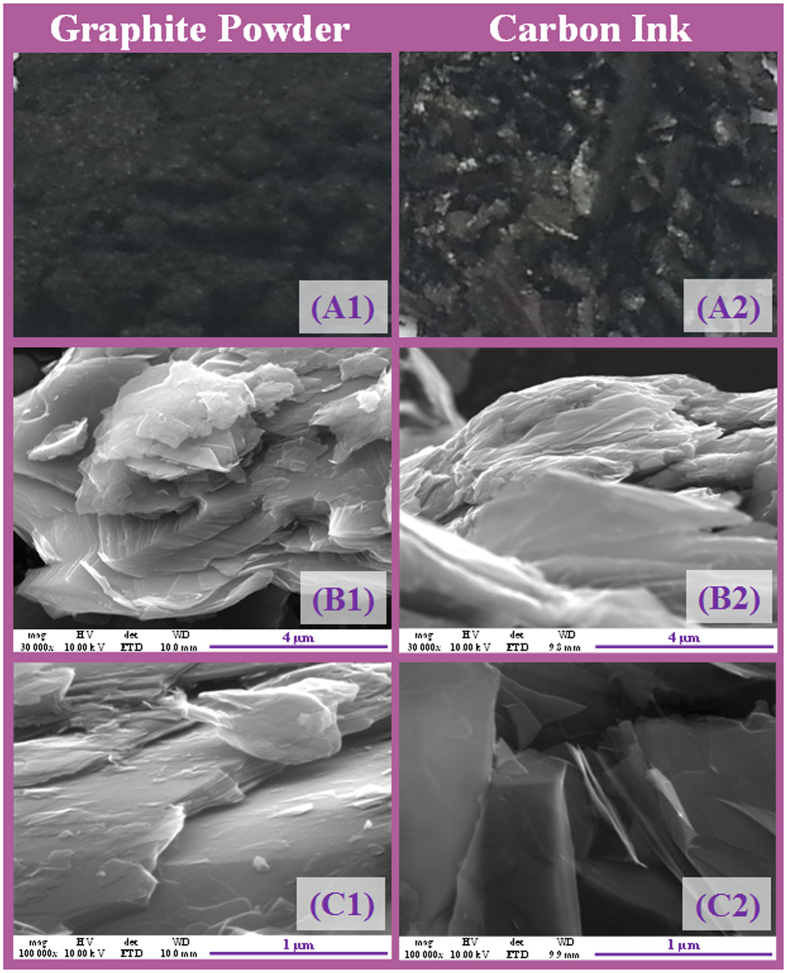
SEM images of graphite (1) and carbon ink (2) of increasing magnifications (**A**–**C**).

The morphology of the final carbon ink was examined by SEM (Fig. 5), comparing the morphologic changes occurring from the original graphite material. The images have shown that graphite powder (Fig. 5A) is clearly different from the conductive ink. In greater magnification, it was possible to see that the particles of graphite (A2) had several compact layers of graphene sheets, in contrast to the carbon ink, where these sheets were extensively separated, suggesting an effective exfoliation of the graphene. EDS analysis also indicated an increasing in the oxygen content when the ink was prepared (compared to the original graphite material), thereby confirming the existence of an oxidative process along the ink preparation.

### Chemical/physical features of the conductive paper

The conductive paper was resistant to mechanical pressure by finger scratching after ink dry. No leaching of graphite particles was evidenced. The resulting electrodes were also left in water with a coloured hydrophilic dye, remaining stable for several days.

**Figure 6:**
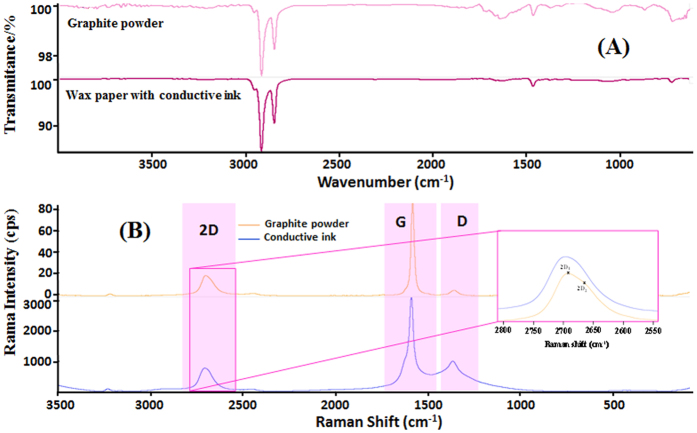
FTIR (**A**) and Raman (**B**) spectra of graphite powder and conductive paper.

The FTIR spectra confirmed the presence of graphite at the outer surface of the conductive paper (Fig. 6) and its subsequent blocking effect to the FTIR incident light source. Cellulose stretching was not evidenced in this, because the graphitic carbon did not allow incident radiation to permeate deeply into the paper. The increased intensity of C–H stretching bands evidenced the presence of the PVC polymer within the graphite-matrix.

The Raman spectra of the conductive paper evidenced the three peaks occurring typically in graphite-based materials. These are known as G, D and 2D peaks^30^. The G peak expressed the C–C stretching of the first order scattering of sp^2^ carbon hybridization and corresponded to the in-plane vibrational mode at E2 g phonons at Brillouin zone centre; the D band originated from a hybridized vibrational mode associated to the double resonance excitation of phonons close to the K point in the Brillouin zone, ~1330 cm^−1^, containing a certain fraction of sp^3^ hybridized carbons that indicated the presence of disorder or defect in the carbon material; the 2D peak^31^ originated from a second-order process, involving two inter-valley phonons near the K point, and was of higher intensity than in graphite. The exact Raman shift and intensity values are indicated in Table 1. Compared to graphite powder, the D band of the conductive paper was of higher intensity, revealing greater disorder in the conductive ink structure. The 2D in the Raman spectra of graphite was represented by two components 2D_1_ and 2D_2_^30^, both more intense than D peak. In contrast, the conductive ink had a single, sharp 2D peak and it was less intense than D peak; according to similarities found in the Raman spectra available in the literature^32^, the main component of such ink may be reduced exfoliated graphene oxide (Fig. 6).

The formation of sp^3^ C–H bonds as well as the breaking of the translational symmetric of sp^2^ C = C, lead to defect in hydrogenated carbon material^31^,^33^. The D band is often referred to as the disorder band or the defect band, which may be used as a measure of the quality of the carbon structures after their modification, through the analysis of the intensity ratio between the D and G bands (I_D_/I_G_), i.e., it is used for quantifying the defect density in the carbon material. The intensity ratio extracted from Table 1 indicated significant disorder, arising from structural defects. The I_D_/I_G_ ratios of the graphite powder and ink were respectively 0.057 and 0.319 cps, reflecting the increase in structural disorder and the occurrence of the evolution of the graphite to graphene. Consequently, this graphene presented more defects than graphite powder. Overall, the obtained Raman spectra indicated that the ink become a graphene-based material, after being submitted to the previously mentioned conditions.

The electrochemical features of the carbon-ink/paper were evaluated by electrochemical impedance spectroscopy (EIS) measurements, making use of [Fe(CN)_6_]^3−^/[Fe(CN)_6]_^4−^ solutions with different concentrations prepared in 1.0 × 10^−2 ^mol/L PBS buffer.

The obtained data was shown as Nyquist plots (Fig. S1), where the resistance to charge transfer (R_ct_) corresponded to the diameter of the observed semicircle. In general, lower R_ct_ indicated quicker electron transfer rates with decreasing Ohmic resistance at the receptor surface. As expected, increasing concentrations of the redox probe yielded lower R_ct_ values. According to the obtained results, an intermediate concentration of 2.5 × 10^−3 ^mol/L of iron probe was selected to proceed with the subsequent electrochemical measurements. This concentration ensured sensitive readings of EIS, thereby favouring the sensitive detection of opposite events, such as electrical blocking or increased conductivity.

### Assembly of the antibody-like material

The antibody-like surfaces were assembled on conductive paper as shown in Fig. 1. First, the conductive ink was oxidized with H_2_SO_4_ to remove unwanted-species hindering the electron transfer rate of the working electrode. This procedure yielded an increased number of hydroxyl (-OH), epoxide (-O-) and carboxyl groups (-COOH), thus leading to an increased negative polarity at the electrode surface that could improve the subsequent electrochemical polymerization processes^34^. The imprinting stage consisted of the electrochemical polymerization of a suitable monomer, in an aqueous solution containing supporting electrolyte (KCl, 0.10 mol/L) and the molecule to be imprinted (CRT). And finally, the imprinted sites were obtained once the template was removed, and these sites should be able to rebind again to another molecule of CRT. The imprinting effect upon the response of the paper-based electrodes was assessed by control materials, prepared similarly but without CRT.

EDOT and DBS were selected as monomers. The electrochemical polymerization of EDOT is well-known, yielding conductive, biocompatible and stable polymers^35^. On the contrary, the electrochemical polymerization of DBS has not been reported yet. DBS is typically employed as an anionic surfactant along with other monomers^36^,^37^, but its structure includes an aromatic ring strongly active towards electrophilic reagents, making its cationic polymerization possible under suitable electrical stimulus. Thus, the possibility of using DBS as regular monomer in electropolymerization to produce a polymeric film was tested herein.

The D/G peak ratio of the conductive paper (0.320) increased up to 0.328 in EDOT-based films but decreased to 0.253 in DBS films, whereas the 2D/G peak ratio (0.245) increased both in EDOT (0.331) and DBS based films (0.374). The Raman shifts were also displaced in all peaks for both EDOT and DBS films; the larger Raman shift differences were observed for DBS films, with stronger evidence for 2D and G peaks.

After polymerizing EDOT or DBS on the conductive ink, the peak intensity ratio I_D_/I_G_ changed in both imprinted materials (EDOT films increased to 0.328 cps, and DBS ones decreased to 0.253 cps), thereby confirming the occurrence of the electrically-induced polymerization. The Raman shifts of D, G and 2D peaks also increased (+9.12, +7.01, and +3.42 cm^−1^, respectively), thereby confirming the polymer formation. These overall changes may also point out that the imprinted DBS polymer showed less disordered structural organization.

### Rebinding to the antibody-like material

**Figure 7:**
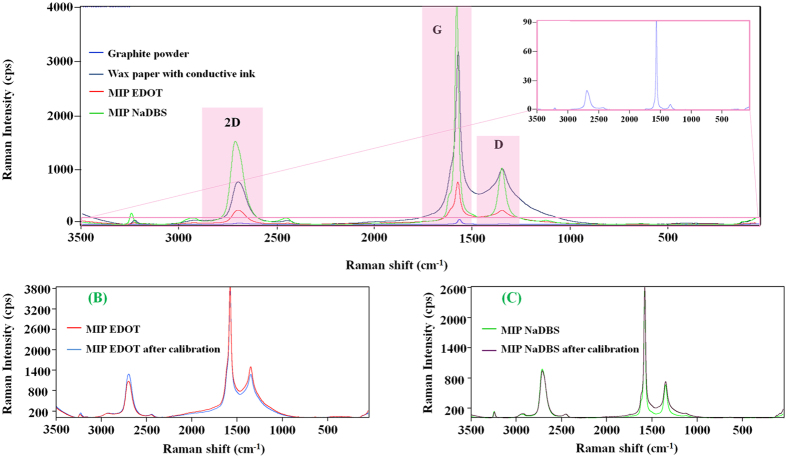
Raman spectra of the antibody-like film on conductive paper (**A**); and a direct comparison before and after calibration of the MI material of EDOT (**B**) or DBS (**C**).

The chemical modifications of the surface morphology occurring after the imprinting stage were characterized by Raman spectroscopy. The corresponding data is indicated in Table 1. As expected, the obtained spectra presented the typical G, D and 2D peaks observed in the conductive paper (Fig. 7), regardless the monomer employed. But relative intensities of D/G and 2D/G peaks varied.

The adsorption of CRT to the antibody-like films was confirmed by Raman analysis of the films that had been calibrated with CRT standard solutions, with a concentration up to 5.0 × 10^−3 ^mol/L. In the calibrated antibody-like EDOT film, all peaks shifted to higher values (D, +0.26 cm^−1^; G, +1.21 cm^−1^; and 2D, +2.14 cm^−1^). Although the absolute Raman intensity increased significantly in the overall spectra, the peak intensity ratio I_D_/I_G_ increased by 0.059 cps and the ratio I_2D_/I_G_ decreased by −0.056. The calibrated antibody-like DBS film showed an opposite behaviour. The presence of CRT within the DBS-polymer matrix yielded Raman negative shifts in D (−0.61 cm^−1^), G (−1.24 cm^−1^) and 2D (−0.91 cm^−1^) peaks. The overall Raman spectra showed lower intensity, but the changes in peak ratio were consistent with the EDOT-observed behaviour: I_D_/I_G_ increased by 0.024 cps and I_2D_/I_G_ decreased by −0.013.

Overall, these results suggested that CRT remained within the imprinted material after calibration, both in EDOT and DBS polymers. A higher amount of CRT was expected to be present in EDOT-based material due to the higher changes in Raman peak intensity ratio observed for this polymer.

### Optimization of the antibody-like assembly

The effect of the relevant variables at the assembly of an antibody-like film was checked by electrochemical impedance spectroscopy (EIS). The electrical resistance was analyzed by Nyquist plots, showing the frequency of the response of the electrode when in contact with any electrolyte. It also indicates the charge transfer resistance (R_ct_) at the electrode surface which is given by the semicircle diameter^38^.

When the sensing layer at the electrodes was chemically modified, the typical charge transfer behaviour of the overall surface changed, thereby confirming the existence of such modifications. The typical charge transfer behaviour is evaluated with a well-known redox probe, such as [Fe(CN)_6_]^3−/4−^.

### CRT concentration at the imprinting stage

The number of effective rebinding positions for CRT depends of the number of CRT molecules entrapped within the imprinted polymer, but such number cannot be as high as to interfere with the polymer growth. This makes the concentration of CRT at the imprinting stage a critical variable. In this work, the imprinted layer was produced by using two different CRT concentrations: 1.0 × 10^−3^ and 1.0 × 10^−2 ^mol/L. Lower concentrations were not tested as the number of template molecules to be imprinted would decrease a lot when compared to the number of monomeric species present.

The results obtained for EDOT monomer are shown in Fig. S2, plotting normalized values for the carbon-ink stage. The correction factor used for this purpose was calculated against the Rct value of the conductive ink. In both concentrations of CRT tested, it was clear that the presence of polymeric EDOT (PEDOT) contributed to decrease the R_ct_ value of the sensing layer. This was attributed to the fact that PEDOT holds conductive properties, thereby increasing the conductivity features of the surface. This effect was less evident for higher concentrations of CRT because the presence of a high number of these species hindered the polymerization of EDOT, yielding a more resistive material. The removal of CRT molecules located at the surface of the polymer was made by oxalic acid (for 1 h), yielding a slight increase of the overall R_ct_. Overall, considering that a biosensor of low resistivity is expected to lead to a higher sensitivity, the concentration of 1.0 × 10^−3 ^mol /L of CRT was selected for further experiments.

### Effect of monomer

**Figure 8:**
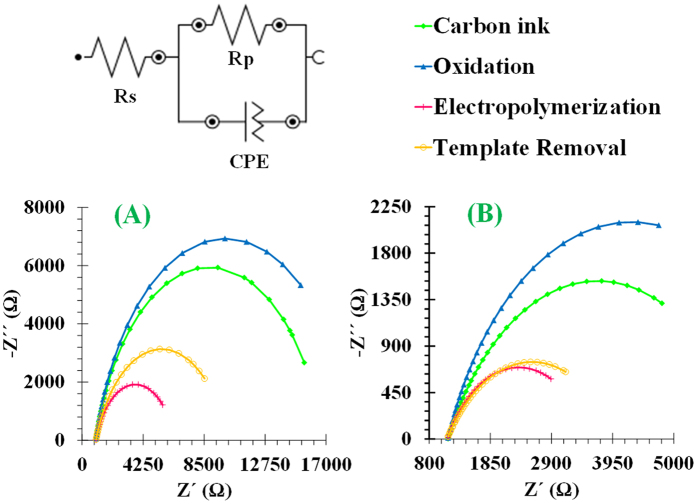
EIS data of the assembly of the antibody-like material. (**A**) sensory layer of DBS; (**B**) sensory layer of EDOT.

The electrical features of the sensory surface changed significantly with the selected monomer (EDOT or DBS). It is important to highlight at this point that the CPE in the electrical circuit is the constant phase element, which can behave as resistance if *n* = 0, capacitance when *n* = 1 or Warburg impedance if *n* = 0.5^39^. In the case of an imprinted DBS polymeric layer, the CPE value changed between 109.25 and 390.09 μF and in EDOT polymer changed from 114.52 to 215.79 μF. Thus, in both cases this element behaved as capacitance, because n ~ 1. The equivalent circuit shown in Fig. 8 was used to analyze the Nyquist plots after each surface modification step at the biosensor fabrication with DBS (Fig. 8A) or EDOT (Fig. 8B).

In both cases, the electrolyte resistance corresponded to 2.5 × 10^−3 ^mol/L of [Fe(CN)_6_]^3−/4−^. The R_s_ values ranged 0.976–1.82 kΩ in DBS sensing layers and 1.11–1.37 kΩ in EDOT sensing materials. Overall, EIS measurements showed that both polymers displayed conductive features, because the original impedance decreased after electrochemical polymerization of EDOT or DBS by chronoamperometry. In addition, and comparing to EDOT sensing films, the DBS-imprinted film yielded higher decrease in resistance (meaning better electrical features) and the subsequent CRT removal promoted a higher resistance change (meaning that a higher number of rebinding positions for CRT are available, and thus better analytical features may be expected).

### Overall electrical performance

The sensors were calibrated by incubating the antibody-like surface in solutions of increasing concentrations of CRT, for 30 minutes, and reading subsequently the EIS electrical features of the resulting surface. The corresponding EIS spectra are shown in Fig. S3, obtained in 2.5 × 10^−3 ^mol/L [Fe(CN)_6_]^3−/4−^ redox probe, and at a standard potential of 0.238 V for imprinted EDOT and 0.222 V for imprinted DBS, with a number of frequencies equal to 50 and an amplitude of 0.01 V in both cases. The frequency range was 0.01–1000 Hz.

**Figure 9:**
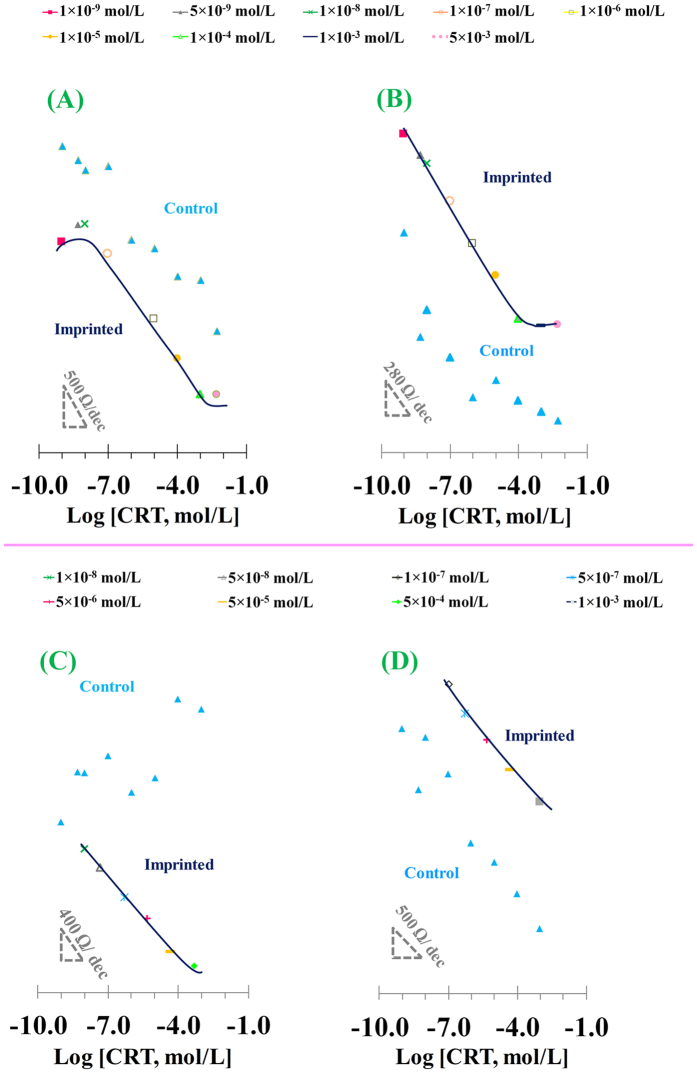
Calibration curve of the imprinted paper-based devices. Imprinted DBS (**A**,**C**) and EDOT (**B**,**D**) based materials in HEPES buffer (**A**,**B**) and urine samples (**C**,**D**). Insets display the corresponding typical calibrations, including also the response of the control materials.

Overall, the R_ct_ values decreased with increasing concentration of CRT. This suggested that the negative redox probe was attracted to the sensory layer when CRT was present, due to their opposite charges. In turn, this also evidenced that CRT was bound to the sensing polymeric films. Figure 9 also shows at the inset the calibration curves plotting R_ct_ against log CRT concentration, for both active and control materials. In general, polymeric films tailored with imprinted positions displayed a linear behaviour for increasing CRT amounts, in contrast to the response of control materials that was mostly random.

In buffer solutions, the imprinted DBS material responded with a linear trend from 1.0 × 10^−8 ^mol/L to 1.0 × 10^−3 ^mol/L, as R_ct_ = −450.12 × log [CRT] + 3943.4, with a regression correlation coefficient of 0.9984 (Fig. 9A). The imprinted EDOT responded similarly, having an R_ct_ = −302.59 × log [CRT] + 2602.5, and a regression correlation coefficient of 0.9979; the limit of detection was 2.15 × 10^−10 ^mol/L and the linear response was up to 1.0 × 10^−4 ^mol/L. All control materials displayed random and uncontrolled behaviour.

The selectivity of the sensory layers for CRT was tested by calibrating the paper-based sensors in real urine samples from healthy volunteer, diluted 10× in HEPES buffer. In general, the resulting EIS calibration curves for DBS (Fig. 9C) and EDOT (Fig. 9D) sensing materials showed good analytical features. The calibration curve of imprinted DBS (Fig. 9C) showed a linear dependence on log [CRT] from 1.0 × 10^−8^ to 5.0 × 10^−4 ^mol /L, with a slope of −376.01 Ω/decade. The calibrations of imprinted EDOT layer displayed linear behaviour from 1.0 × 10^−7^ to 1.0 × 10^−3 ^mol/L, with a slope of −452.4 Ω/decade. Control materials showed once again a random behaviour, thereby confirming that the response was mainly controlled by the rebinding of CRT to its imprinted positions on the polymeric matrix.

Overall, no interference from co-existing species present in real urine is expected to exist in the analysis of real samples, suggesting that these sensors would be capable of providing selective and specific readings of CRT in real samples. Moreover, a calibration using the imprinted DBS films was performed in synthetic urine (without CRT, to compare its performance to that made in real urine (Fig. S6). BSA was also included is this assay, in order to have a protein in the calibration matrix and evaluate its possible interference. The resulting calibrations showed the sensor under synthetic urine displayed similar behaviour to that of the real urine. In this regard, it seems that all components present in the synthetic urine are not capable of promoting significant changes in the calibration output.

### Application and reusability

Spiked urine samples were analyzed by the previously described sensors. For this purpose, a urine sample from health individual was collected and spiked in several levels of CRT. The errors linked to the known amount of CRT found in the system ranged from 3.9 to 6.5% in the case of the imprinted DBS film, with an average relative standard derivation of 1.1%. In the case of the imprinted EDOT material, the relative error ranged from 9.1 to 15.4%, with an average relative standard deviation of 3.3%. These results indicated that both materials displayed good accuracy and precision, being DBS-based paper sensors the ones providing better relative errors.

Real urine analysis was conducted by multiple standard addition and subsequent use of the Gran’s method to estimate the original concentration of the CRT in the urine sample^40^ (Fig. S5). In this, the linear plot of 10^Rct/S^ *versus* known carnitine concentration (added) crossed the *x* axis at the unknown CRT concentration (present in the sample). The CRT concentration in the original sample was estimated in 0.06 μmol/L (or 9.67 ng/mL), which is compatible with the levels of a healthy individual.

The reason for such good analytical performance is perhaps a consequence of the very similar EIS data obtained in consecutive calibrations of the same sensor (Fig. S4). The fact that consecutive calibrations yielded very similar features, pointed out the possibility of reusing such sensors. But not many calibrations could be made consecutively. This meant that CRT was standing on the surface and hindering the possibility of recovering the original signal. So, the CRT sensory layers were tested for an electrochemical cleaning, carried out by performing consecutive CV assays in HEPES buffer. These assays were made between −0.2 V and +1 V, at a potential scan-rate of 0.05 V/s with a number of crossings 10 (5 successive CV cycles). The efficiency of this cleaning process was tested by EIS, in the same redox couple [Fe(CN)6]^3−/4−^, at a standard potential of 0.22 V, and using a number of frequencies equal to 50 scans and a sinusoidal potential peak-to-peak with amplitude 0.01 V, in the 0.01–1000 Hz frequency range.

It was very interesting to observe that the results showed that both antibody-like films had better features in terms R_ct_ after electrochemical cleaning. The DBS based sensor presented a slope increase of ~70 Ω/decade (~20%) and the limit detection was 1.93 × 10^−9 ^mol/L; the most surprising results were those obtained with EDOT films, yielding slopes of ~450 Ω/decade (80% higher), for a limit of detection of 2.25 × 10^−8 ^mol/L, saturating the electrical response by 5.0 × 10^−3 ^mol/L CRT.

In general, the electrochemical cleaning ensured not only reusability but also improved the observed analytical features. These features remained stable after 3 consecutive procedures of alternated calibration and cleaning and valid for more than a month.

## Conclusions

The use of low cost wax to make the paper hydrophobic was simple and effective, regarding the electrical requirements behind electrochemical sensing. The conductive ink proposed was prepared by quick and low cost procedures, displaying excellent conductivity, thermal stability and good adhesion to the cellulose support. The tailoring of an antibody-like film for CRT was successfully achieved by simple electrochemical procedures and the film displayed high sensitivity/selectivity for rebinding CRT, even in complex matrix composition such as urine samples.

The limit of detection obtained also allows its practical application to the analysis of biological fluids. Moreover, detection limits of these biosensors are better, when compared to other commercial methods. For instance, the coupled enzyme assays in L-carnitine assay kits (from Sigma-Aldrich) have typical sensitivities of 1612 ng/mL (colorimetric) or 161 ng/mL (fluorimetric) of carnitine, while ELISA KITs display common sensitivities of 78 ng/mL. Herein, MIP EDOT and MIP NaDBS sensors showed limits of detection of 3.63 ng/mL and 0.31 ng/mL, respectively, also over a wide concentration of linear response range. Considering that a healthy individual may have <161.2 ng/mL of CRT in urine, the described sensors may be applied to real samples of healthy or diseased individuals. In addition, despite the low cost and simplicity of preparation, the paper-based electrodes can be reused by several times before discard.

Overall, the simple construction, low cost and reusability suggest that these electrodes could have commercial viability for screening CRT or other molecules in POC. In addition, the application of the conductive ink proposed herein for the first time may be extended to prepare conductive films on different supports (such as glass, ceramics, or PET). Also, the present methodology is versatile considering that its application to different fields of research and knowledge may be anticipated. Plastic antibodies may be tailored for a wide range of target biomolecules or the homemade carbon ink may be applied to the screen-printing technology making use of different support materials, including paper.

### Methods and Materials

#### Apparatus

Polymeric film assemblies and electrochemical measurements were made in a potentiostat/galvanostat equipment from Metrohm, Autolab, PGSTAT302N, computer controlled by NOVA software. A cellulose paper support (3.3 × 1.0 cm) made conductive with conductive ink was used as working electrode; a platinum wire as counter electrode; and a double-junction Ag/AgCl electrode as reference.

The thermal behaviour of conductive ink was evaluated in the thermogravimetry (TG)/differential thermal analyzer (DTA) *Exstar TG/DTA 7200.* The resistivity of the ink was measured by Fluke 175 True RMS multimeter. A Sonorex digitec sonicater, *Bandelin*, was used to promote the dissolution of the solids and homogeneity of the reacting solutions.

FTIR surface analysis of solid materials was made in a Nicolet iS10 spectrometer from Thermo Scientific coupled to a smart attenuated total reflectance (ATR) sampling accessory of germanium contact crystal, also from *Nicolet*. Raman analysis was performed using Thermo Scientific DXR Raman equipment coupled to confocal microscopy with 50× lenses (dark field/bright field) and 532 nm laser. The digital image of the contact angle was acquired by a digital camera Samsung PL150.

#### Reagents

All chemicals were of analytical grade and ultrapure Milli-Q laboratory grade water (conductivity < 0.1 μS.cm^−1^) was employed. Cellulose paper was obtained from Fanoia. Graphite powder, phosphate buffered saline (PBS) tablets, 3,4-ethylenedioxythiophene (EDOT), 4-(2-hydroxyethyl)-1-piperazineethanesulfonic acid (HEPES) and oxalic acid dehydrate (OAc) were purchased to Sigma-Aldrich; dodecylbenzenesulfonic acid sodium salt 88% (DBS) to Acros Organics; *N,N*-dimethylformamide (DMF) to VWR; poly(vinylchloride) carboxylated (PVC-COOH) to Fluka; potassium hexacyanoferrate III (K_3_[Fe(CN)_6_]) and potassium hexacyanoferrate II trihydrate (K_4_[Fe(CN)_6_]·3H_2_O) to Riedel-de-Haën; CRT hydrochloride, and potassium chloride (KCl) to Merck; and sulphuric Acid (H_2_SO_4_) to Scharlau. Urea was obtained from Fagron, creatinine to Fluka, magnesium chloride to Riedel-de-Haën, calcium chloride to Purified, sodium dihydrogen phosphate to Scharlau, potassium sulphate and sodium chloride to Panreac, ammonium chloride to Merck and bovine serum albumin to Sigma.

#### Solutions

Stock standard solutions of CRT were prepared with a concentration of 1.0 × 10^−3 ^mol/L, prepared in 1.0 × 10^−2 ^mol/L HEPES buffer, pH 7.0. Less concentrated solutions (calibrating standards) were prepared by accurate dilution of the previous solution in the same buffer. Potentiostatic electropolymerization was made in solutions of 0.01 mol/L EDOT or 0.1 mol/L DBS, prepared in 0.1 mol/L KCl as supporting electrolyte in water. Electrochemical assays were performed in a solution containing 2.5 × 10^−3 ^mol/L K_3_[Fe(CN)_6_] and 2.5 × 10^−3 ^mol/L K_4_[Fe(CN)_6_], prepared in PBS buffer, pH 7.0.

### Production of the conductive paper

The working electrode was constructed by cutting small pieces of cellulose paper (1.5 × 1.0 mm). Each piece was hydrophobized with wax, by heating up to 95 °C, for 3 hours. The paper was cooled to room temperature (~20 min). The external surface of the waxed paper was then made conductive by applying the conductive ink by the doctor Blade technique. The ink was prepared simply by mixing graphite powder doped with 15% PVC-COOH in DMF. The ink casted on the paper was dried at 55 °C, for 1 h.

The thermal behavior of conductive ink was monitored by TG analysis in the temperature range 35–1000 °C, for a heating rate of 5 °C/min, in a nitrogen atmosphere of 200 mL/min. Similar experiments were made with control materials, graphite powder and PVC-COOH, making use of the same experimental conditions.

### Surface analysis of the conductive paper

The contact angle of the resulting conductive paper was measured for a drop of a pink dye solution placed on it. A digital image captured by a camera allowed measuring the interior angle formed on the surface, making use of the tangent line to the drop interface at the apparent intersection of all three interfaces. The PowerPoint program of Windows was used for to obtain the contact line and also find the corresponding interior angle.

FTIR analysis of the conductive paper was made directly on the ATR accessory. All spectra were collected under room temperature/humidity control after background correction. The number of scans was 32 for each samples and background. X-axis represented wavenumber, ranging 4000–600 cm^−1^, and Y-axis % transmittance. Raman analysis was conducted after focusing the material on the optical microscope with a 50× lens. The spectra were collected with 8 mW power and 50 μm pinhole aperture. Automatic fluorescence and photoblishing corrections were made.

### Assembly of the antibody-like film

Before use, the carbon surface of the conductive paper was subjected to an electrochemical oxidation, by imposing 5 successive cycles, in 0.5 mol/L of H_2_SO_4_, from −0.2 to +1.5 V, at a scan-rate of 50 mV/s. Antibody-like films were prepared by eletropolymerization of EDOT or DBS, made by chronoamperometry (+0.9 V for 240 s), in a solution containing 1.0 × 10^−3 ^mol/L CRT (template), 1.0 × 10^−1 ^mol/L KCl (supporting electrolyte) and 1.0 × 10^−2 ^mol/L EDOT or 1.0 × 10^−1 ^mol/L mol/L DBS. After polymerization, the template was removed by incubation of the films (on the conductive paper) in 0.50 mol/L oxalic acid, for 1 h. Control materials were prepared in parallel, by excluding the template from the procedure.

### Electrochemical assays

Cyclic voltammetry (CV) experiments were conducted in HEPES buffer, pH 7.0, in 2.5 × 10^−3 ^mol/L K_3_[Fe(CN)_6_]/K_4_[Fe(CN)_6_]. The potential was scanned from −1.0 and +1.0 V at a scan rate of 50 mV/s, and with 10 crossing points (5 successive CV cycles). EIS measurements were conducted in the same redox couple [Fe(CN)6]^3−/4−^ at a standard potential of 0.22 V, for 50 frequency values from 0.01–1000 Hz and a sinusoidal potential peak-to-peak amplitude of 0.010 V. The impedance data was fitted to a suitable electrochemical circuit using the ANOVA software.

The response of each working electrode to increasing concentrations of CRT was evaluated by incubating the electrode for a fixed period of time, in concentration values ranging from 1.0 × 10^−8^ to 5.0 × 10^−3 ^mol/L. These solutions were prepared in 1.0 × 10^−2 ^mol/L of HEPES, pH 7.0. This incubation period was followed by CV and/or EIS readings made in the previously indicated conditions. Selectivity studies were performed by electrochemical assays with K_3_[Fe(CN)_6_] and K_4_[Fe(CN)_6_] in the same buffer, after incubating the sensing layer in spiked urine samples. The initial urine sample solution was adjusted to 1.0 × 10^−8 ^mol/L, and then this concentration was increased up to a maximum value of 5.0 × 10^−3 ^mol/L.

The analytical application was tested in real samples (urine, diluted 1:10 in HEPES buffer). This was done by calibrating the electrodes in solutions with blank urine and analyzed after real samples spiked with known amounts of CRT. Different electrodes were used for the calibration and sample analysis procedure, for which the electrical output signal was always considered relative to blank (buffer signal prior to the incubation with the first standard).

## Additional Information

**How to cite this article**: Tavares, A. P. M. *et al*. Conductive Paper with Antibody-Like Film for Electrical Readings of Biomolecules. *Sci. Rep.* **6**, 26132; doi: 10.1038/srep26132 (2016).

## Supplementary Material

Supplementary Information

## Figures and Tables

**Table 1:** Analytical data extracted from the collected Raman spectra of the several materials.

**Material**	**Monomer**	CRT* **mol/L**	**Raman intensity**			**Raman Shift**			**Peak ratio**	
			***2D Peak***	***G Peak***	***D Peak***	***2D Peak***	***G Peak***	***D Peak***	***I***_***D***_***/I***_***G***_	***I***_***2D***_***/I***_***G***_
Graphite powder	—	—	19.68	91.64	5.22	2693.58	1565.83	1337.93	**0.057**	**0.215**
Conductive paper	—	—	780.59	3184.93	1017.78	2697.00	1572.84	1347.05	**0.320**	**0.245**
Antibody-like	EDOT	—	258.09	780.70	256.31	2696.58	1574.52	1347.65	**0.328**	**0.331**
Antibody-like	EDOT	5 × 10^−3^	1062.73	3862.63	1493.69	2698.72	1575.73	1347.91	**0.387**	**0.275**
Antibody-like	DBS	—	1528.25	4085.84	1034.29	2711.64	1579.64	1349.25	**0.253**	**0.374**
Antibody-like	DBS	5 × 10^−3^	940.13	2606.84	722.85	2710.73	1578.40	1348.64	**0.277**	**0.361**

^*^Calibration of the material with CRT, up to a concentration of 5.0 × 10^−3 ^mol/L.
