# Supplementary material for: Conductive Paper with Antibody-Like Film for Electrical Readings of Biomolecules
Source: Sci Rep. 2016 May 23;6:26132. doi: 10.1038/srep26132 (PMC4876510; doi:10.1038/srep26132)
Supplement: Supplementary Information [file srep26132-s1.pdf]

SUPPLEMENTARY SECTION

Conductive Paper with Antibody-Like Film for Electrical Readings of Biomolecules

Ana P. M. Tavares[1], Nádia S. Ferreira[1], Liliana A.A.N.A Truta[1], M. Goreti F. Sales[1,*]

[1]*BioMark-CINTESIS/ISEP, School of Engineering, Polytechnic Institute of Porto, Portugal*

TG features of the carbon-ink. TG analysis allowed studying the thermal decomposition behavior of the different ink compounds, as depicted in Figure 4 (main paper). The differential gravimetric data of the conductive ink (Figure 4B) showed little mass loss up to 201.0 ºC, of ~1.06 %. The significant mass loss occurred in a two-stage behavior, from 201.0 to 360.1 ºC and after that up to 488.6 ºC, corresponding to two endothermic peaks centered at 273.2 ºC and 441.1 ºC, respectively. Considering that the first mass loss corresponded to 8.92 % and the second one to 3.81 %, this overall mass loss (12.73 %) was assigned to the degradation of PVC-COOH. A 15% theoretical mass loss could be achieved if all PVC-COOH was degraded, which compared well with an experimental mass decrease of 12.73%. Moreover, a third mass loss was evident between 488.6 ºC and 1000º C. This mass loss was small (3.37 %) and occurred over a large temperature range (~511.4 ° C). It was probably related to a small thermal decomposition of the graphite powder along this high temperature range, as evidenced by the thermogram of pure graphite powder presented in Figure 4A.

The thermal assay of pure PVC-COOH (Figure 2C) also presented two mass losses at temperatures very close to those observed in the conductive ink: 218.6 ºC to 371.7 ºC (corresponding to 60.96 % mass); and 371.7 ºC to 519.5 ºC (corresponding to 26.89 % mass). Both ranges corresponded to endothermic events, centered at 274.9 ºC and 432.3 ºC, respectively. The mass loss in the first temperature range was assigned to the evolution of HCl to form polyenes, leading to the competition of two reactions: decomposition for

producing volatiles, mainly benzene, or intermediate cross-linked material decomposing at higher temperatures to produce pyrolysis and more volatile products (aromatics)[38,39]. The mass loss in the second temperature range was attributed to the evolution of toluene and methylated aromatics, coming from the decomposition of polyenes and meaning that a substantial quantity of hydrogen chloride and aromatic groups could be present[38,39].

Compared to the pure PCV-COOH, the temperature ranges of decomposition of the conductive ink shifted negatively in 17.6/11.6 ºC for the first significant mass loss, and 11.6/30.9 ºC for the second mass loss. The maximums peaks of degradation corresponding to these ranges occurred at about the same temperatures, with maximum shift of 8.8 ºC. These results revealed the presence of PVC-COOH within the conductive ink and that the presence of graphite powder yielded little effect upon the thermal degradation of this polymer. Moreover, the conductive ink offered high thermal stability, remaining stable up to 201 ºC.

Electrical changes caused by the redox probe concentration. The electrochemical behavior in terms of response of these conductive paper was evaluated by EIS measurements of different concentrations solutions of $K_3[Fe(CN)_6]$ and $K_4[Fe(CN)_6]$ in 1.0×10^{-2} mol/L PBS. For this study the same electrode was used in the measurement in different concentrations of redox probe. The corresponding Nyquist plots are shown in Figure S1.

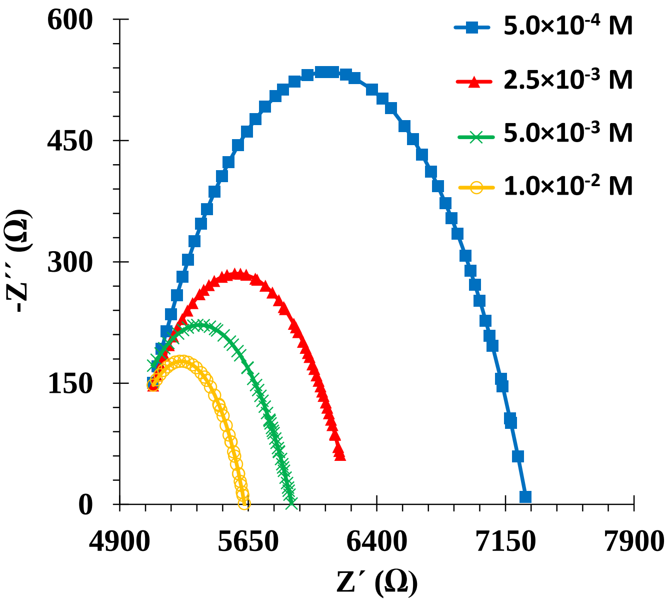

Figure S1 EIS data for different iron redox probe concentrations prepared in HEPES buffer.

In general, the Ohmic resistance increased when the concentration of $K_3[Fe(CN)_6]$ and $K_4[Fe(CN)_6]$ decreased. An intermediate concentration was selected in this study, 2.5×10^{-3} mol/L $K_3[Fe(CN)_6]$ and $K_4[Fe(CN)_6]$, as a compromise between having high electrical transfer rates for a low concentration of iron redox probe.

CRT concentration at the imprinting stage.

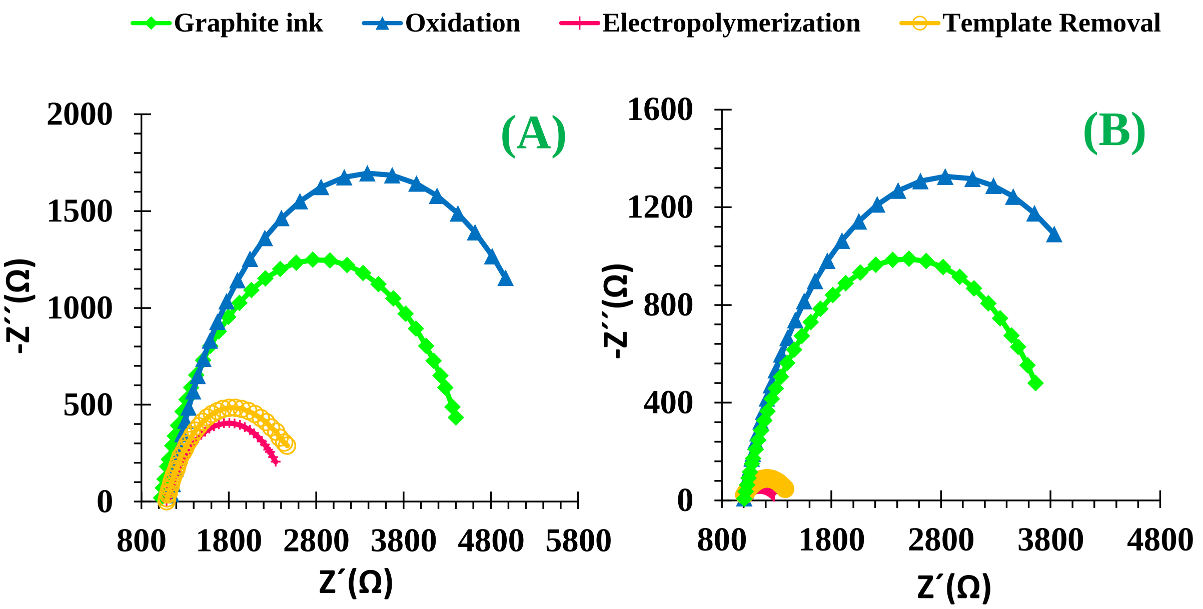

Figure S2 – EIS data for CRT concentrations at the imprinting stage equal to (A) $1.0{\times}10^{-2}$ mol/L or (B) $1.0{\times}10^{-3}$ mol/ L (EDOT was the monomeric unit of the polymer), and the corresponding electrical circuit.

Overall electrical performance.

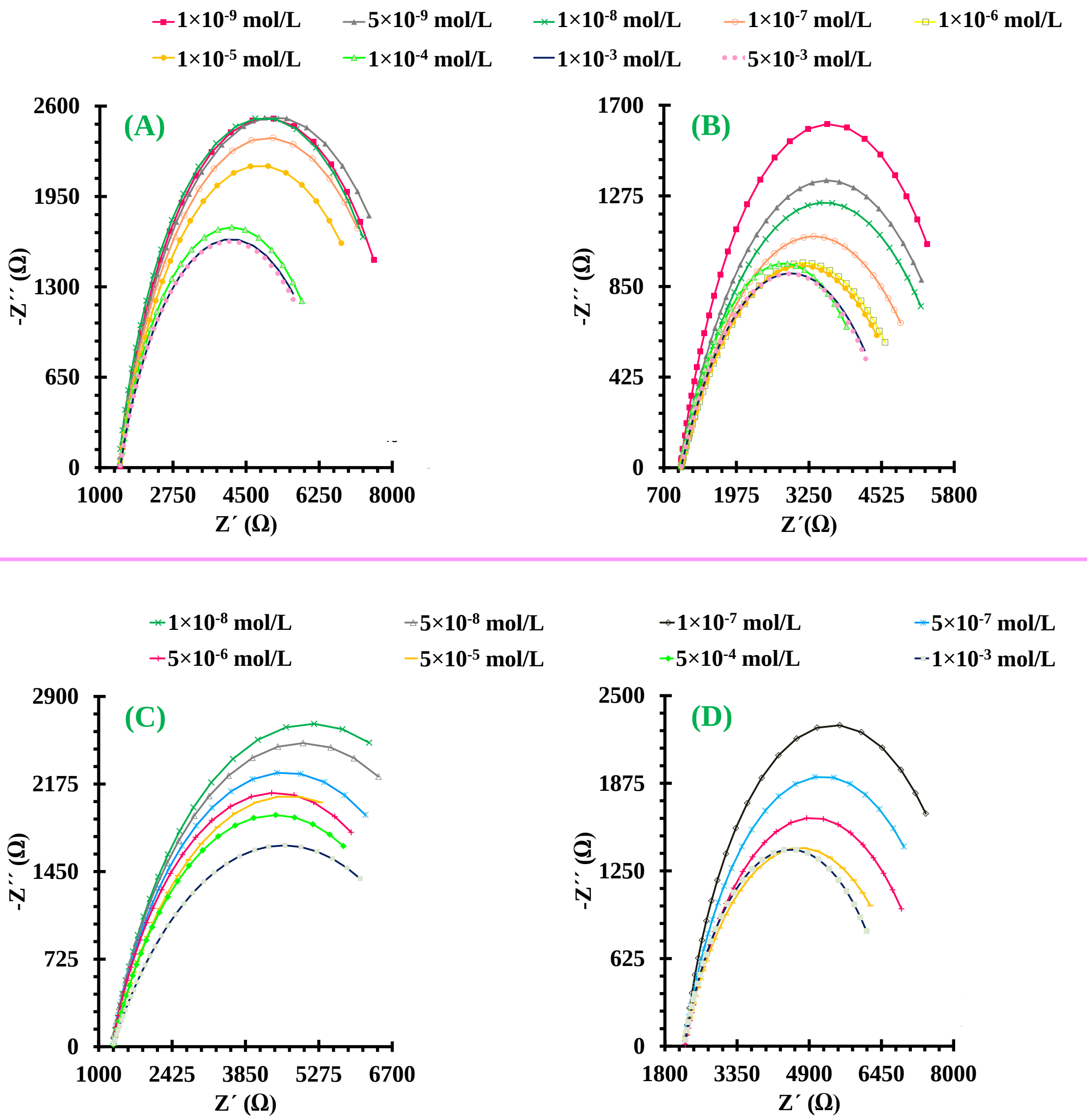

Figure S3 – EIS spectra obtained by calibrating the imprinted paper-based devices. Imprinted DBS (A,C) and EDOT (B,D) based materials in HEPES buffer (A,B) and urine samples (C,D).

Consecutive calibrations.

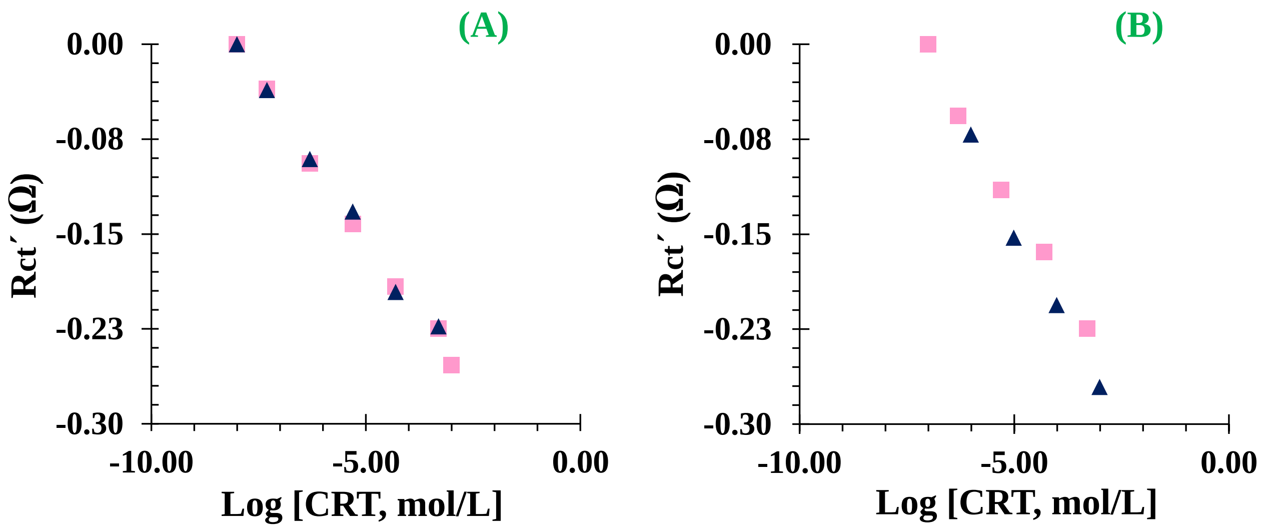

Figure S4 – Consecutive calibrations in serum solutions of the antibody-like polymeric sensors prepared with DBS (A) and EDOT (B) monomers.

Gran's Method of multiple standard addition. This method allows determining the unknown concentration and minimizing matrix effects, such as varying ionic strength or the presence of interfering ions. The plot of $10^{Rct/S}$ *versus* the concentration of carnitine added gave a straight line where the *x* axis intercept indicated directly the concentration of the unknown on the analysed sample (carnitine already present in the urine real sample before spiking with different standard levels).

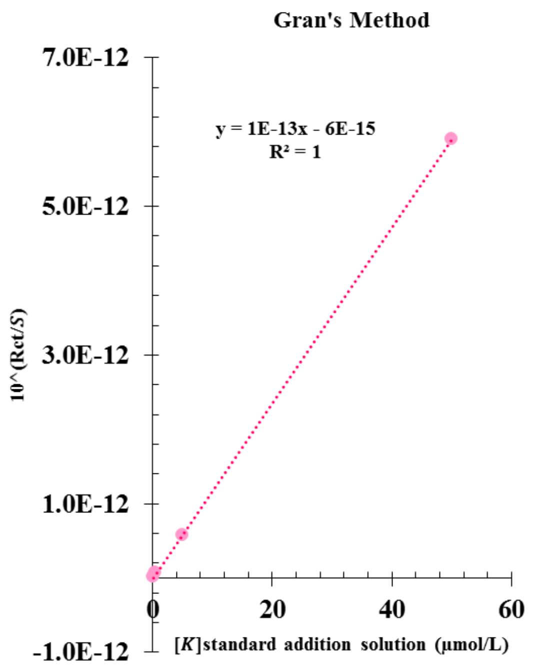

Figure S5 – Calibration curve obtained for a CRT sample. S corresponds to the slope of the experimental calibration; Rct the charge transfer resistance of each spiked level; and [k] the known concentration of carnitine present in the each level of spiked sample.

The data points seem superimposed for lower values, but this results from the use of a wide concentration range using a logarithmic scale in the normal calibration and using this data to have a Gran's plot. The estimated CRT concentration in urine was 0.06 µmol/L (or 9.67 ng/mL), a value that lies within the concentration range of healthy individuals.

Synthetic urine. The selectivity of the sensory layers for CRT was also tested by calibrating the paper-based sensors in synthetic urine with the overall composition: 5.0×10^{-6} M urea, 5.3×10^{-6} M creatinine, 9.8×10^{-7} M magnesium chloride, 6.8×10^{-7} M calcium chloride, 3.2×10^{-6} M sodium dihydrogen phosphate, 3.3×10^{-6} M ammonium chloride, 3.9×10^{-6} M potassium sulphate, 6.5×10^{-6} M sodium chloride and 3.0×10^{-8} M bovine serum albumin (BSA).The calibration of the sensors under synthetic urine is presented in Figure S6 and indicates a similar behaviour to that under real urine. Thus, negligible interference from a controlled urine composition, where BSA was added as protein source, is unexpected.

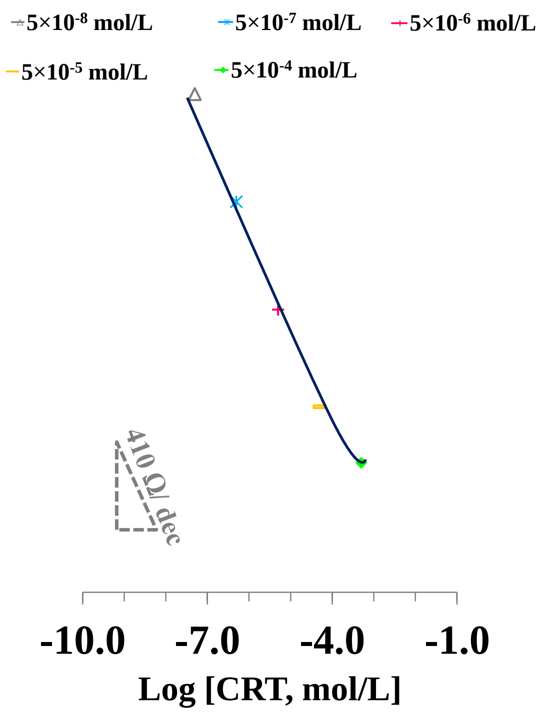

Figure S6 – Calibration curve obtained for CRT in synthetic urine.

Analytical Parameters

Table S1- Further bioanalytical Parameters.

**Material**	**Monomer**	**Limits**		**Precision**			**%CV**	**Reproducibility**
		LOD (mol/L)	*LOQ (mol/L)*	*Linearity (mol/L)*	*Intra-day N=3*	*Inter-day N=3*	*Intraday N=3*	*Fabrication N=4*
Antibody -like	EDOT	2.25×10^{-8}	7.51×10^{-8}	1.0×10^{-7}–5×10^{-3}	1.29±0.09	1.45±0.29	7.34%	1.04±0.05
Antibody -like	DBS	1.93×10^{-9}	6.42×10^{-9}	1×10^{-8}–5×10^{-4}	1.85±0.05	1.93±0.12	3.09%	0.51±0.18
